# Sleep disturbances in hospitalized children: a wake-up call

**DOI:** 10.1007/s00431-024-05660-x

**Published:** 2024-07-03

**Authors:** Pia Burger, Lindsay M.H. Steur, Jorinde A.W. Polderman, Jos W.R. Twisk, Robert Lindeboom, Reinoud J.B.J. Gemke

**Affiliations:** 1 Department of Pediatrics, Emma Children’s Hospital, Amsterdam UMC, Amsterdam, The Netherlands; 2 Amsterdam Reproduction and Development Research Institute, Amsterdam, The Netherlands; 3 Department of Anaesthesiology, Amsterdam UMC, Amsterdam, The Netherlands; 4 Department of Clinical Epidemiology and Data Science, Amsterdam Public Health, Amsterdam UMC, Amsterdam, The Netherlands

**Keywords:** Sleep, Hospitalization, Discomfort

## Abstract

Although sleep is essential for (recovery of) health, it is adversely affected by hospitalization, due to disease discomfort, environmental noise, and care routines, causing reduced sleep and increased disturbances. This study evaluates factors affecting sleep quality and quantity in hospitalized children and compares inpatient sleep with sleep at home. Using an observational, prospective study design, we assessed sleep in hospitalized children aged 1–12 years, admitted to a tertiary center, and compared this with home 6–8 weeks after discharge. We measured total sleep time (TST), sleep onset latency (SOL), wake after sleep onset (WASO), sleep efficiency, awakenings, and subjective sleep quality, using actigraphy, sleep diaries, and PROMIS questionnaires. We explored an array of sleep-disturbing factors. Regression analyses identified key determinants affecting sleep patterns, while mixed linear models compared sleep in hospital to sleep at home. Out of 621 eligible patients, 467 were invited, and 272 (58%) consented to participate. Key determinants of sleep included pain, number of previous admissions, (underlying) chronic illness, and environment-, staff-, and disease-related factors. Parents reported lower perceived sleep quality in the hospital compared to at home, 97-min (SE 9) lower TST, 100-min (5) longer WASO, more difficulties with falling asleep, lower sleep satisfaction, and more awakenings. Actigraphy outcomes revealed shorter TST (20 min (6)), but better sleep efficiency and fewer awakenings in the hospital. *Conclusion*: Sleep in hospital was compromised in comparison to sleep at home, primarily due to disturbances related to treatment, environment, and staff. These findings underscore the necessity and potential of relative simple interventions to improve sleep quality and minimize sleep disturbances in hospitalized children.

**Supplementary Information:**

The online version contains supplementary material available at 10.1007/s00431-024-05660-x.

**Table:** 

**What is known:** * • Sleep is essential for maintenance of health and recovery from disease, yet sleep is substantially affected by hospitalization.*	
**What is new:** * • We assessed multiple nights of in-hospital sleep of a large heterogeneous group of admitted children and compared this with 7 days of objective home sleep data as reference.*	

## Introduction

Sleep is crucial for normal growth and development and for maintenance of health, while impacting memory, cellular repair, brain development, and hormonal balance [1–5]. For children, proper sleep includes adequate duration, uninterrupted sleep, age-appropriate naps, and alignment with natural circadian rhythms [6, 7]. Hospitalized children face substantial sleep disturbances including disease-related pain and discomfort, environmental factors like noise and light, psychosocial factors (e.g., anxiety, fatigue, or parenting-related), and frequent visits of nursing and (para)medical staff [8–16]. These disturbances lead to shorter sleep duration, increased awakenings, and longer sleep onset latency compared to home [8, 10, 17–20]. Research methods vary, including questionnaires, diaries, video, and actigraphy, but so far often miss to combine subjective and objective aspects of sleep. Since subjective and objective measures comprise different constructs, combining both ensures a more nuanced understanding of sleep [21, 22]. Furthermore, most studies involve small samples [8, 10, 11, 17, 18, 20, 23–30], and few examine larger groups [19, 31].

The primary objective of this study was to identify which factors (environment-, disease-, staff-, patient-, and treatment-related factors) most significantly influence sleep during hospitalization. Additionally, the prevalence of different sleep disturbances is investigated. Together, this provides an insight into potential amendable sleep disturbers, subsequently enabling the development of interventions to improve sleep [32]. The secondary objectives focused on comparing sleep during hospitalization to at home and examined the evolution of sleep throughout the hospital stay.

## Methods

### Ethics

The study was approved in accordance with the Declaration of Helsinki by the hospitals’ Medical Ethics Review Committee (ref# NL71596.018.20, date of approval: June 9, 2020). Parent written informed consent was obtained for all participants.

### Setting

A prospective, observational study on sleep in hospitalized children was conducted at Emma Children’s Hospital, Amsterdam UMC, a tertiary care center in the Netherlands serving newborns to 18-year-olds across specialized units, including three general wards and one acute admission unit.

### In- and exclusion criteria

Inclusion criteria comprised that participants had to be admitted for at least one night (8 PM–8 AM), aged between 1 and 12 years. Young infants (< 1 year of age) were excluded because they often do not have a stable circadian rhythm yet. Children from age 12 upwards were excluded as their sleep habits may change substantially due to puberty. Moreover, the proxy assessment of sleep by parents that we used in our study becomes hampered by puberty [33–35]. Children in the Pediatric Intensive Care Unit (PICU) were excluded due to frequent invasive procedures, mechanical ventilation, and sedatives affecting sleep patterns. Similarly, children within 24 h post-operative were also excluded. Additionally, those with moderate to severe developmental disorders (IQ<85), known sleep disorders, and limited Dutch fluency were excluded.

### Study procedure

Recruitment ran from March 2021 until December 2023, using daily medical reports and nurse consultations to identify potential participants. The research team explained the study and provided written information to potential participants from 10 AM to 5 PM on weekdays. After obtaining informed consent, participants wore an actigraph, and parents completed a sleep diary and questionnaire daily until discharge or for a maximum of 7 nights. The sleep diary and questionnaire used after the first night also included references to habitual sleep at home during the month prior to hospitalization. Six to eight weeks post-discharge, a follow-up kit with an actigraph and a sleep diary was sent home, after confirming convenience by phone. Home sleep was monitored for seven consecutive nights.

### Measurements

#### General information

Essential (demographic) information was gathered, including age, sex, the number of previous hospital admissions, the existence of chronic diseases, reason for admission, vital signs, type of treatment, and medication.

#### Actigraphy

Sleep patterns of hospitalized children were tracked using a wrist-worn actigraph (ActiGraph wGT3X-BT), a non-intrusive device that objectively measures sleep/wake patterns through wrist movements. Its data, showing a sensitivity of 0.82–0.91 and specificity of 0.47–0.81, are close to polysomnography results for estimating wake and sleep periods [36, 37]. The actigraph could be worn on either wrist [38]. Data were recorded in 1-min epochs.

**Fig. 1:**
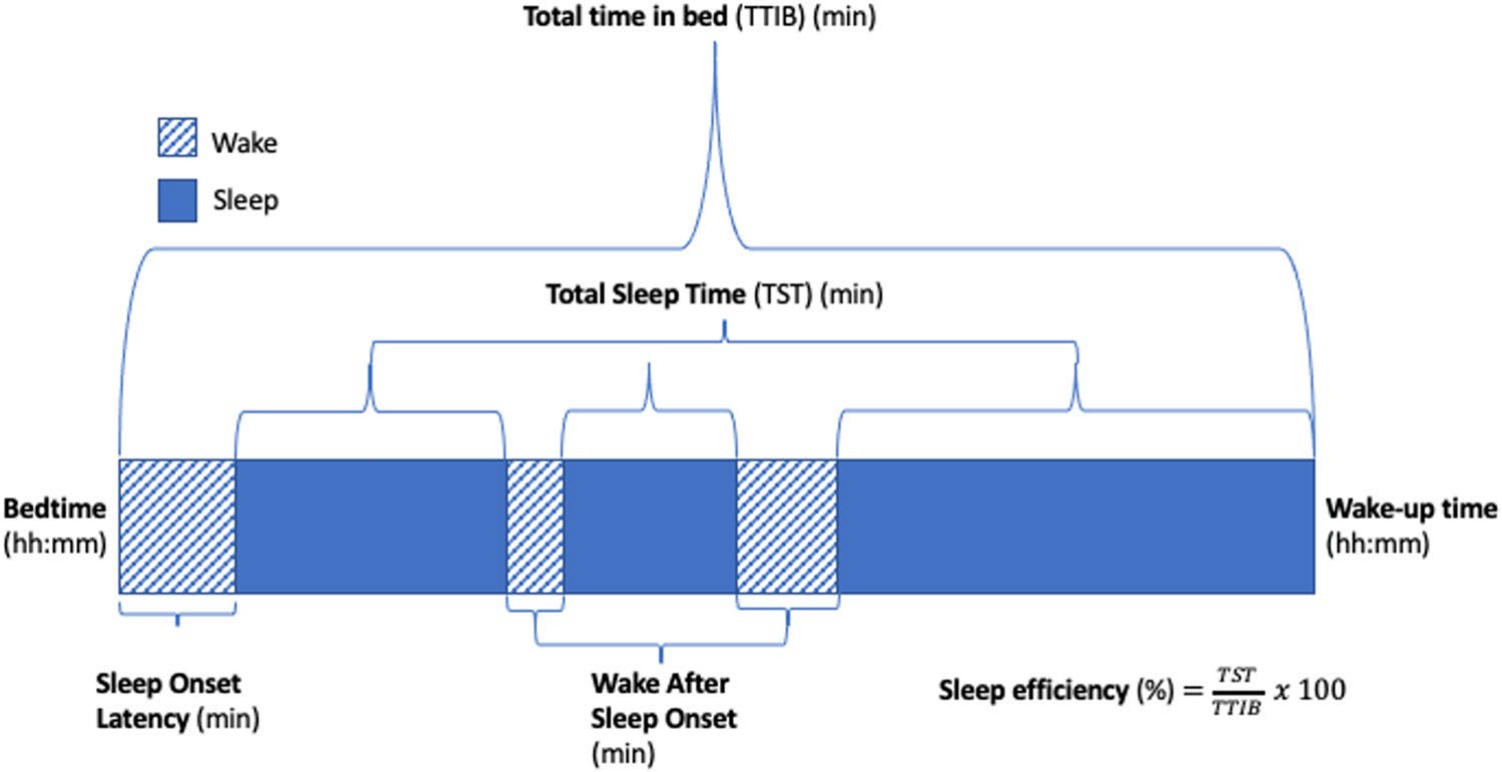
Explanation of sleep variables

Actigraphy data were processed with Actilife version 6.13.3. The following sleep outcomes were generated by the use of the validated Sadeh algorithm [38]: total sleep time (TST), sleep onset latency (SOL), sleep efficiency, number of awakenings, and wake after sleep onset (WASO) (see Fig. 1, and for a definition of each variable see [14]). These variables were calculated based on bedtimes and wake times provided by parents in a sleep diary.

#### Sleep diary

A sleep diary was included to capture parent-reported sleep quantity [39]. The diary provided information about bed/wakeup times, SOL, WASO, number of awakenings, and daytime napping. From these measures, TST and sleep efficiency were derived. Sleep diary outcomes from the first night after inclusion in the study (hospital setting) were compared to habitual sleep at home during the month prior to hospitalization (home setting).

#### Questionnaire

The questionnaire was adapted from one used in a previous study on adult inpatients at Amsterdam UMC, modified to focus on the parents of hospitalized children, rather than the patients themselves [40]. No further changes were made. The questionnaire included measures for assessing sleep quality and sleep-disturbing factors. Details of the different components are provided in the subsequent paragraphs.

#### Sleep quality

A selection of items from the Dutch-Flemish Patient-Reported Outcomes Measurement Information System (PROMIS), version 1.0, sleep disturbance item bank (Short Form 8a) was used to measure sleep quality. Specifically, five of the eight items from this item set were chosen, along with a sixth item from the complete PROMIS sleep disturbance item bank, deemed most suitable for measuring sleep disturbance in hospitalized patients [41, 42]. The six items included three positive items (sleep quality, satisfied, rested) and three negative items (restless, difficulty, feeling lousy), rated on a 5-point scale (ranging from “very poor/not at all” to “very good/very much”). To measure the differences in sleep quality between the hospital and home settings, three items (sleep quality, satisfied, difficulty) were presented with reference to both the previous night’s sleep at the hospital and habitual sleep at home during the month prior to hospitalization. Scores for positive aspects were inverted, making higher scores indicating lower sleep quality. Given the study’s adapted timeframe for assessing hospital and habitual home sleep (i.e., the previous night in hospital and the month prior to hospitalization, respectively, rather than the past seven nights as in the original PROMIS questionnaire), traditional PROMIS *T*-scores were not calculated. Instead, raw summary scores for the five sleep disturbance item set (ranging from 0 to 20) were used to describe the overall sleep disturbance experienced by the patients. Cronbach’s alpha was calculated at an acceptable 0.77.

#### Disturbing factors

Potential factors that could impact sleep were recorded, such as the frequency of nightly vital checks, pain levels (numeric rating scale (NRS) pain 0–10 scale), the type of room occupied (double or single), daily activities, and staff-related, environment-related, disease-related, and patient-related disturbances that might occur during hospitalization.

Staff-related factors involved awakenings for vital checks, staff noise, room changes, medicine administration, diaper changes, and nutrition assistance. Environment-related factors comprised medical equipment noise, noise from other patients/parents, lighting, uncomfortable beds, temperature, and miscellaneous sounds. Disease-related factors included symptoms, sleeping positions, wounds, and pain. Patient-related reasons were the unfamiliar environment, fear, stress, toileting needs, appetite, inactivity, nightmares, and sadness. Treatment-related reasons, as noted in the electronic patient record, encompassed interventions like intravenous drips, blood platelet transfusions, intravenous medications, low-flow oxygen, night Optiflow, catheters, and other treatment-related disturbances.

### Data analysis

Data analysis included four key parts: (1) analysis to explain sleep during hospitalization based on data from the first night after inclusion, (2) describing the prevalence of sleep disturbances in hospital, (3) comparing sleep from the first night after inclusion to habitual sleep at home, and (4) examining the course of sleep throughout the hospital stay.

Linear regression or negative binomial regression for count data was used to identify factors that most significantly explain sleep during hospitalization, with data transformations on the dependent variables in case of non-compliance with assumptions. This initial analysis concentrated on data from the first night after inclusion, because of decreasing numbers due to varying length of stay. The goal was to find the best-fitting model to explain sleep quality with minimal variables using backward stepwise selection based on Akaike’s Information Criterion [43]. Dependent variables included actigraphy-measured TST, SOL, WASO, number of awakenings, sleep efficiency, parent-reported bedtimes, wake times, and PROMIS total score. Independent variables considered included disturbing factors (environment-, staff-, disease-, patient-related), age, sex, number of previous hospital admissions, length of hospital stay, presence of chronic disease, reason for hospitalization (medical vs surgical), ward type (acute admission unit vs regular care unit), and NRS pain scores.

The second analysis focused on the prevalence of parent-reported sleep disturbances attributed to staff, environment factors, disease-related factors, patient-related factors, and treatment-related disturbances and are presented as frequencies and percentages for each sleep outcome (SOL, WASO, or wake-up time).

The third analysis focused on comparing sleep quality and quantity between hospital and home settings. The first hospital night sleep after inclusion was compared to habitual sleep at home for perceived sleep quality (PROMIS items). Sleep quantity during hospitalization was compared with both sleep outcomes prior to hospitalization, and with follow-up at home 6–8 weeks post-discharge and included TST, bedtime, wake-up time, and actigraphy-measured variables including TST, SOL, WASO, number of awakenings, and sleep efficiency. Linear mixed models were developed, using the setting (hospital vs home) as the independent variable, and included a random intercept and a random slope for the setting at the patient level. All models were subjected to checks for normality of residuals, homoscedasticity, and multicollinearity. Where residuals did not demonstrate approximately normal distributions, transformations of the dependent variables were considered.

The final analysis was used to examine the evolution of sleep outcomes throughout the hospital stay. Similarly to the third analysis, mixed linear models were used, using a random intercept and a random slope for each night at the patient level.

Descriptive statistics incorporated means and standard deviations, medians and interquartile ranges, and frequencies and percentages, depending on the nature and distribution of each variable under consideration. Comparisons of the demographic data for patients who were not approached versus those who participated, as well as those who participated in follow-up versus those who did not, have been conducted To address missing data, Multiple Imputation by Chained Equations (MICE) was employed [44] (see Table 1 for the imputed variables). Actigraphy 6 weeks after discharge was not imputed (NA=113). Analyses were performed in R, version 4.3.1, using packages MASS, nlme, lme4, glmTMB, mice, and lubridate.

**Table 1:** Baseline child and medical characteristics

Child and medical factors		Study participants (*n* = 272)
Age (in years), mean (sd)		6.4 (3.6)
Female sex, *n* (%)		119 (43.8)
Having an underlying chronic disease, *n* (%)		97 (35.6)
Number of previous admissions, median [IQR]		2 [0–4]
Surgical specialties (as opposed to medical specialties)^a^, *n* (%)		145 (53)
Pre-study hospital nights, median [IQR]		1.5 [1–3]
Admitted for an exacerbation of a chronic disease		80 (29.4)
Type of ward, *n* (%)	Acute admission unit	87 (32)
	Regular care units	185 (68)
Total days of admission at inclusion, median [IQR]		2.5 [2–4]
Mother rooming-in (as opposed to father)^b^, *n* (%)		189 (70)
Single bedroom^b^, *n* (%)		245 (90)
Number of vital checks during nighttime, median [IQR]		2 [1–4]
Pain^b^, median [IQR]	Nighttime	1 [0–4]
	Daytime	3 [1–6]
Type of medication^b^, *n* (%)	Sleep-inducing	2 (0.7)
	Anxiolytic	2 (0.7)
	Analgesic	60 (22)
Imputed missing data:	Previous hospital admissions (NA = 28), underlying chronic disease (NA = 19), PROMIS items (each NA = 9), bedtime home (NA = 7), SOL home (NA = 13), awakenings home (NA = 35), WASO home (NA = 52), wakeup home (NA= 11), daytime nap home (NA=12), hospital parent reported SOL (NA = 12), hospital parent-reported WASO (NA = 16), hospital parent-reported awakenings (NA=9), hospital parent-reported daytime nap (NA = 16), PROMIS items (max NA = 5), pain score night (NA =34), actigraphy hospital (NA = 29)	

Abbreviations: *IQR*, interquartile range; *sd*, standard deviation

^a^Surgical specialties included general surgery, plastic surgery, urology, ear/nose/throat surgery, and orthopedic surgery

^b^Data from the first night after inclusion

## Results

**Fig. 2:**
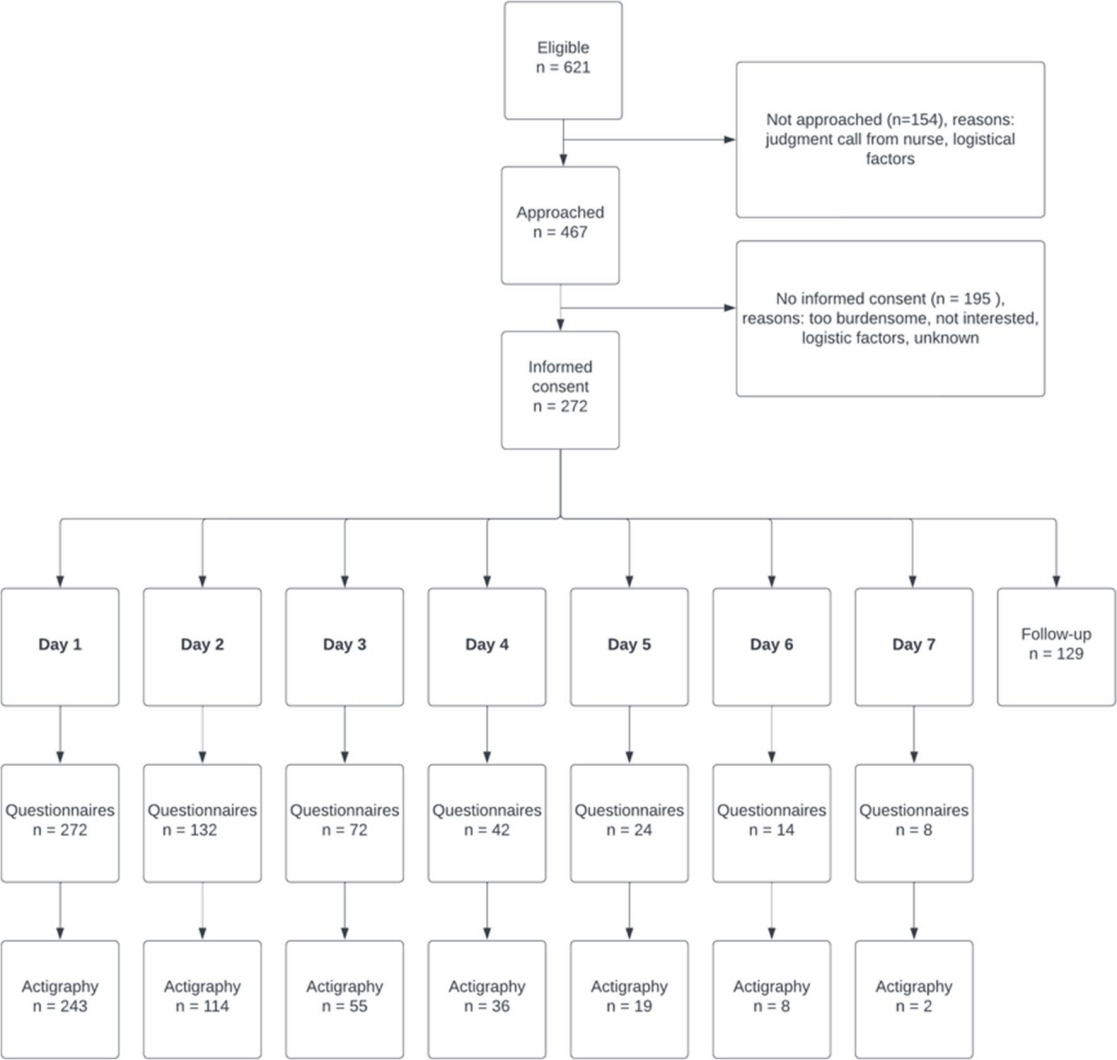
Patient enrollment

Of 621 eligible patients, 467 were invited to participate, with 154 not invited primarily due to nurses deeming inclusion too burdensome. Those who were not approached for participation did not differ in age or sex, yet they were more frequently on the acute admission ward than other wards (50% vs 32%, respectively). Informed consent was obtained from 272 patients [58%]. The primary reason for declining was the perceived study burden. Among the 272 participants (mean age 6.4 [SD 3.6]; 44% female), 243 wore an actigraphy device for at least one night. The median pre-study hospital stay was 1.5 [IQR 1–3] nights. The average participation duration was 2.0 [SD 1.5] nights. Surgical specialties were predominant (145 [53%], Table 1). Eighty patients [29%] were admitted for a chronic disease exacerbation, and 87 [32%] received treatment in the AAU. Follow-up home sleep registrations were completed for 129 [47%] patients (Fig. 2) (see supplementary information, Table 1 for a comparison between patients participating in follow-up and those who did not).

### Factors explaining hospital sleep

Key factors affecting sleep quality and quantity among patients are shown in Table 2. Most frequently recurring factors included (1) pain score during the night, which was retained in the models explaining bedtime, TST, SOL, WASO, sleep efficiency, and PROMIS total score, and (2) having a chronic (underlying) disease, which was retained in the models explaining bedtime TST, awakenings, and sleep efficiency. The explained variance for the different sleep variables ranged from 0.03 to 0.29, with the PROMIS total score showing the largest explained variance.

**Table 2:** Different models with factors explaining sleep-related variables

Sleep variables	Included factors	Effect size (95 CI, *p*-value)
Total sleep time	Surgical reason for hospitalization	−19.2 (−42.0; 3.5, *p* > 0.05)
	Admitted for chronic illness*	22.4 (−47.2; 2.36, *p* > 0.05)
	Environmental-related disturbances*	22 .8 (−0.01; 45.5, *p* = 0.05)
	Pain score night	−4.2 (−8.51; 0.11, *p* = 0.05)
Sleep onset latency	Pain score night	−0.07 (−0.17; 0.02, *p* > 0.05)
Number of awakenings	Age	0.02 (−0.03; −0.003, *p* < 0.05)
	Presence of chronic disease*	0.12 (−0.02; 0.28, *p* > 0.05)
	Admitted for chronic illness*	−0.12 (−0.28; 0.03, *p* > 0.05)
	Treatment-related disturbances*	−0.15 (−0.34; 0.04, *p* > 0.05)
Bedtime^a^	Admitted for chronic illness*	0.26 (−0.50; −0.01, *p* < 0.04)
	Pain score night	0.08 (−0.13; −0.04, *p* < 0.001)
	Environmental-related disturbances*	−0.16 (−0.40; 0.06, *p* > 0.05)
	Age	0.08 (0.05; 0.11, *p* < 0.001)
Wake-up time	Ward type*	−11.3 (−26.1; 3.5, *p* > 0.05)
	Staff-related disturbances*	15.4 (1.3; 29.6, *p* < 0.05)
	Treatment-related disturbances*	33.4 (12.5; 54.3, *p* < 0.005)
Wake after sleep onset	Pain score night	5.0 (1.8; 9.3, *p* < 0.005)
	Age	6.4 (−8.7; −4.0, *p* < 0.001)
Sleep efficiency	Age	0.7 (0.37; 1.11, *p* < 001)
	Presence of chronic disease*	−2.24 (−5.16; 0.36, *p* > 0.05)
	Pain score night	−0.77 (−1.2; −0.26, *p* < 0.05)
PROMIS total score	Number of previous hospital admissions	1.2 (0.40; 2.0, *p* < 0.05)
	Pain score night	0.42 (0.25; 0.59, *p* < 0.001)
	Environmental-related disturbances*	1.2 (0.40; 2.0, *p* = 0.05)
	Disease-related disturbances*	2.26 (1.36; 3.17, *p* <0.001)
	Patient-related disturbances*	1.2 (0.39; 2.03, *p* < 0.05)

*Dichotomous (0 or 1), ^a^Yeo-Johnson transformation

### Prevalences of sleep-disturbing factors

Among 272 patients, 252 patients (93%) experienced disturbances related to sleep onset, awakenings, or waking up, primarily due to staff-related (54%), disease-related (41%), and patient-related (41%) reasons. Additionally, 85.5% were affected by treatment-related disturbances. Sleep onset disturbances were reported by 141 (56%) patients. Patients attributed sleep onset disturbances to staff (22%), environment (31%), disease (28%), and patient (20%) factors. A total of 205 patients (75%) reported disturbances related to night awakenings. Forty-two percent of the patients mentioned staff-related disturbances, 26% environment, 31% disease-related, and 28% patient factors. The most frequently cited disturbances for sleep onset and awakenings were lights, staff members for vital checks or medicine, noises from medical equipment, and pain. Upon waking, 141 patients (52%) mentioned one or more disturbances. Patients attributed wake times to staff (25%), environment (21%), disease (10%), and patient factors (10%).

### Comparison of sleep between hospital and home

Table 3 compares the PROMIS items of sleep disturbance in the hospital to the month prior to hospitalization. Parents perceived their children’s sleep quality worse in hospital compared to home (mean item score difference −0.7 points), were less satisfied (−0.4 points), and reported that their child had more difficulty falling asleep in the hospital than at home (−0.4 points).

**Table 3:** Parent-reported sleep quality at home (retrospectively) and in the hospital (*n* = 272)

	Very good (0)^c^	Good (1)^c^	Fair (2)^c^	Poor (3)^c^	Very poor (4)^c^	Mean score (SE)^a^	Difference (SE)^b^
My child’s sleep quality was							
Hospital	**25** (9)	**119** (44)	**95** (35)	**25** (9)	**8** (3)	2.3 (0.1)	−0.7 (0.1)
Home	**97** (36)	**138** (51)	**30** (11)	**6** (2)	**1** (0)	1.5 (0.2)	
	Very much (0)	Quite a bit (1)	Somewhat (2)	A little (3)	Not at all (4)		
I was satisfied with my child’s sleep							
Hospital	**40** (15)	**128** (47)	**55** (20)	**31** (11)	**18** (7)	1.9 (0.1)	−0.4 (0.1)
Home	**82** (30)	**129** (48)	**31** (11)	**18** (7)	**12** (4)	1.4 (0.2)	
My child had difficulty falling asleep							
Hospital	**125** (46)	**57** (21)	**28** (10)	**45** (17)	**17** (6)	1.6 (0.2)	−0.4 (0.1)
Home	**151** (56)	**70** (26)	**34** (13)	**12** (3)	**5** (2)	0.7 (0.3)	
My child woke up rested	**14** (5)	**102** (37)	79 (29)	**51** (19)	**26** (10)	1.9 (0.1)	
My child’s sleep was restless	**87** (32)	**83** (31)	**60** (22)	**33** (12)	**9** (3)	1.2 (0.1)	
My child woke up lousy when waking up	**164** (60)	**69** (26)	**21** (8)	**17** (6)	**1** (0)	0.6 (0.1)	

^a^Differences indicate hospital minus home item scores on a 0–4 point scale. ^b^As per the mixed linear model, with random intercept and slope, *p*<0.001 for all comparisons. ^c^Number of participants (percentages)

Table 4 presents a comparison of sleep quantity measures (sleep diary and actigraphy) during hospitalization with parent-reported sleep diary outcomes for the month prior to hospitalization (*n*=272) and actigraphy measurements during follow-up at home 6 to 8 weeks post-discharge (*n*=129). Parent-reported TST was 97 (9) min less in the hospital, bedtime delayed by 34 (14) min, wake-up time 11 (4) min earlier, and WASO extended by 101 (5) min, leading to a 6% (0.8) drop in sleep efficiency compared to habitual sleep prior to hospitalization. Additionally, parents noted 1.2 (0.1) more awakenings in the hospital and 7 (3) min longer daytime naps. When comparing hospital sleep with post-discharge sleep, actigraphy indicated a 20-min (6) lower TST in the hospital compared to home. However, a 14-min (5) lower WASO, a 2% (0.8) higher sleep efficiency, and less awakenings (7 times (0.9)) were noted in the hospital (all comparisons *p*<0.05).

**Table 4:** Sleep quantity and timing measures

Sleep outcome	Hospital mean (SE)	Home mean (SE)	Mean difference (SE)
Comparisons between hospital and parent-reported sleep prior to hospitalization (*n* = 272)			
Bedtime (hh:mm)	21:03 (27.8)	20:27 (40.2)	33.8 (14.1)
Wake-up time (hh:mm)	7:29 (6.9)	7:18 (10.4)	10.5 (3.7)
Wake after sleep onset (min)	213.5 (9.0)	112.7 (13.6)	100.8 (4.6)
Total sleep time (min)	446 (15.2)	543 (23.8)	96.9 (8.9)
Sleep efficiency (%)	82.8 (1.7)	88.8 (2.5)	5.0 (0.8)
Number of awakenings (*n*)	3.3 (0.2)	2.0 (0.3)	1.2 (0.1)
Daytime nap (min)	43.7 (6.7)	36.7 (9.4)	6.9 (2.9)
Comparisons between hospital and 6–8 weeks post-discharge with actigraphy (*n* = 129)			
Wake after sleep onset (min)	98.7 (8.9)	112.6 (14.0)	13.9 (5.3)
Total sleep time (min)	462 (11.1)	483.0 (17.1)	20.8 (6.3)
Sleep efficiency (%)	81.9 (1.4)	79.6 (2.2)	2.3 (0.8)
Number of awakenings (*n*)	14.0 (1.3)	21.0 (2.0)	7.1 (0.8)

*p*<0.05 for all differences

### Course of sleep throughout the hospital stay

Actigraphy showed a significant decline in sleep efficiency throughout hospitalization by 0.76% (0.3) per day, an increase in SOL by 2.3 (0.8) min, more frequent awakenings by 0.77 (0.8) times, and longer WASO by 5.4 (2.3) min.

## Discussion

The primary objective of this study was to identify the determinants that most effectively explain disturbances in sleep quality and quantity during hospitalization. We found that multiple factors are associated with the various sleep variables, including environmental-, staff-, and disease-related disturbances; pain scores; (underlying) chronic disease; and age. The connection between age and sleep aligns with other studies, indicating that younger children require more sleep [7, 45], while older children tend to have shorter SOL, reduced WASO, and consequently better sleep efficiency [46]. Pain was an influencing factor for most sleep outcomes, impacting sleep quality, TST, bedtime, WASO, SOL, and sleep efficiency. Research in adults has shown that insufficient sleep can increase pain sensitivity [47], and other studies have linked pain with reduced sleep duration in hospitalized children [11, 29, 48–51]. Enhancing sleep could therefore improve children’s pain management and potentially reduce the need for analgesics. Conversely, better pain management might lead to improved sleep. Additionally, compromised sleep is tied to lower patient satisfaction [52], which can increase emotional distress, potentially leading to depression and anxiety [53]. This can create a cycle where emotional distress leads to poorer sleep, and vice versa [54].

About 93% of patients reported sleep disturbances, mainly due to staff interactions. Perhaps illustrative for the inadvertent impact of daily routine is the finding of disturbances from early wake-up times for breakfast delivery by general facilities staff, even when unnecessary (e.g., for tube-fed children). Previous research highlights healthcare professionals’ limited awareness of sleep’s role in health and recovery [55]. This study emphasizes the importance and potential of educating all healthcare staff on factors affecting children’s sleep quality to potentially improve recovery outcomes.

We also compared sleep during hospitalization to sleep at home. Parents reported worse sleep quality for their child in the hospital, with later bedtimes, longer WASO, shorter TST, and more awakenings. Actigraphy data showed significant later bedtimes and lower TST, but also showed contradictions to the parent-reported measures, showing fewer awakenings, reduced WASO, and better sleep efficiency in the hospital. These paradoxical findings might stem from the differing nature of the comparisons made: actigraphy measurements at home were averaged over 7 days, in contrast to a single night’s measurement in the hospital, which could vary significantly. The results revealed that sleep significantly deteriorated during hospitalization, with actigraphy showing an increase in SOL, more frequent awakenings, and longer WASO. This suggests that averaging actigraphy over hospitalization days could potentially have revealed more unfavorable actigraphy sleep outcomes in the hospital compared to home. However, due to the decreasing number of children participating each night, we opted against averaging sleep variables. Another explanation may be that sleep patterns may take some time to return to normal following a hospital admission, which might have resulted in a smaller difference between sleep quality in hospital and at home. Additionally, actigraphy might have overestimated sleep efficiency [56], and reliance on parents’ reports during hospitalization could be influenced by their preoccupation with their child’s illness or their own lack of proper sleep, potentially affecting the accuracy of their responses. Moreover, parents might not be fully aware of the number of awakenings at home if children sleep in their own rooms.

The strengths of this study include its large and heterogeneous patient sample, along with the use of both objective and subjective sleep measurements. Additionally, it compares in-hospital sleep with at-home sleep as a paired reference. A limitation is the in-hospital analysis only includes data from the first day of inclusion, a decision driven by early discharge policy resulting in a relative short length of stay with a rapidly decreasing sample size over time. Another constraint is the absence of EEG measurements to differentiate between true sleep and restfulness, a significant consideration since hospitalized children often remain in bed due to illness. EEG measurements, being invasive and challenging to implement in home settings, make actigraphy a viable and valid alternative. Another limitation is that subjective sleep and pain scores are reported by parents instead of being self-reported by children. Sleep disturbances could be wrongly attributed to pain if a child wakes up for reasons other than pain, or a child may be in pain that is not recognized by the parents. Furthermore, this study’s findings are derived from a single-center investigation, necessitating additional (multi-center) studies for confirmation. Lastly, the study does not account for factors such as socioeconomic status (SES) or ethnicity, despite evidence suggesting their correlation with children’s sleep duration [57, 58].

## Conclusion

Sleep in the hospital was significantly disturbed, predominantly by treatment-, environment-, and staff-related disturbances. Parents reported worse sleep quality, lower satisfaction, and more difficulties related to their child’s sleep in the hospital setting compared to home. The results underscore the complex interplay of factors affecting sleep in the hospital, highlighting both the need and the potential for targeted, relative simple interventions to improve sleep quality and reduce sleep disturbances during hospitalization.

### Supplementary information

ESM 1 (PDF 124 kb)
