# Supplementary material for: Sleep disturbances in hospitalized children: a wake-up call
Source: Eur J Pediatr. 2024 Jul 3;183(9):4063–72. doi: 10.1007/s00431-024-05660-x (PMC11322318; doi:10.1007/s00431-024-05660-x)
Supplement: Supplementary file 1 — (PDF 124 kb) [file 431_2024_5660_MOESM1_ESM.pdf]

Supplementary information for

Sleep disturbances in hospitalized children: a wake-up call

Pia Burger, MD[1,2]; Lindsay M.H. Steur, MD, PhD[1]; Jorinde A.W. Polderman, MD, PhD[3]; Jos W.R. Twisk, PhD[4]; Robert Lindeboom, PhD[4]; Reinoud J.B.J. Gemke, MD, PhD[1,2]

[1] Department of Pediatrics, Emma Children's Hospital, Amsterdam UMC, Amsterdam, Netherlands.

[2] Amsterdam Reproduction and Development research institute, Amsterdam, Netherlands.

[3] Department of Anaesthesiology, Amsterdam UMC, Amsterdam, The Netherlands.

[4] Department of Clinical Epidemiology and Data Science, Amsterdam Public Health, Amsterdam UMC, the Netherlands

Table 1 A comparison between participants with vs without home actigraphic measurements

Child and medical factors	Participants with home measurements (n = 129)	Participants without home measurements (n = 143)	P-value for difference
**Age** (in years) – mean (sd)	6.8 (3.6)	6.8 (3.6)	NS
Female **sex** – n (%)	61 (47)	58 (41)	NS
**Having an underlying chronic disease –** n (%)	89 (69)	87 (61)	NS
**Number of previous admissions** – median [IQR]	1 [0-3]	2 [1-4]	0.018
**Surgical specialties (as opposed to medical specialties)[a]–** n (%)	73 (57)	72 (50)	NS
**Pre-study hospital nights** – median [IQR]			
**Admitted for an exacerbation of a chronic disease**	28 (21)	52 (36)	0.011
**Acute Admission Unit** - n (%)	47 (36)	40 (28)	NS
**Total days of admission at inclusion –** median [IQR]	2 [2-4]	3 [2-4]	NS
**Number of Vital Checks during nighttime–** median [IQR]	2 [1-3]	3 [1-4]	NS
**Pain[b] –** median [IQR]			
Nighttime	2 [0-5]	1 [0-3.5]	NS
Daytime	3 [1-6]	3 [0-5.5]	NS

Abbreviations: IQR, interquartile range; sd, standard deviation

[a] Surgical specialties included: general surgery, plastic surgery, urology, ear/nose/throat surgery and orthopedic surgery.

[b] Data from the first night after inclusion
